# Beyond lifestyle interventions: exploring the potential of anti‐obesity medications in the UK

**DOI:** 10.1111/cob.12248

**Published:** 2018-04-24

**Authors:** J. P. H. Wilding

**Affiliations:** ^1^ Obesity and Endocrinology Clinical Research University of Liverpool & Aintree University Hospital Liverpool UK

**Keywords:** Anti‐obesity medications, obesity, weight management

## Abstract

In the UK, over one‐quarter of the adult population have obesity (body mass index ≥30 kg m^−2^). This has major implications for patients’ health and the National Health Service. Despite published studies showing that significant weight loss can be achieved and maintained in primary care, and guidance from the National Institute for Health and Care Excellence, weight management services are inconsistently implemented. This may be due primarily to workload and financial constraints. There is also a lack of belief that specialist weight management services and anti‐obesity medications (AOMs) are a viable alternative to bariatric surgery for long‐term maintenance of weight loss. This article discusses the challenges facing obesity management and explores the reasons for the lack of investment in AOMs in the UK to date. The aim of this article is to identify whether the newer AOMs, such as naltrexone/bupropion and liraglutide 3.0 mg, are likely to perform better in a real‐world setting than current or withdrawn AOMs. In addition, it considers whether the equitable provision of specialist weight management services and future clinical trial design could be improved to help identify those individuals most likely to benefit from AOMs and, thus, improve outcomes for people with obesity in the UK.

## Introduction

In 2015, the UK had the highest level of obesity in Western Europe (26.9%), ahead of countries such as France (15.3%), Germany (23.6%) and Spain (16.7%) 1. The economic implications are substantial due to both direct healthcare costs and the loss of productivity and life years 2.

Although there are guidelines for the prevention and management of obesity in the UK 3, 4, 5 several challenges present when implementing these in clinical practice. The foundation of obesity management is diet and exercise, supported by behaviour change (i.e. lifestyle intervention), but many people are unable to achieve a clinically meaningful weight loss (defined as ≥5% after 1 year of treatment 6) with this approach alone 7. In 2013, the National Health Service (NHS) Commissioning Board recommended the introduction of multidisciplinary weight management services for people with severe and complex obesity, including for those being considered for bariatric surgery, which include specialist oversight, training in behaviour change techniques and psychological support 8. These services should be made available to people with obesity for whom lifestyle interventions have not been successful 8. Many people accessing these multidisciplinary services report benefits from this level of engagement and find that they are better able to make permanent lifestyle modifications in ways that lead to improvements in weight 8. However, the provision of weight management services is implemented inconsistently across the UK 8. Bariatric surgery is recommended for those with a body mass index (BMI) >40 kg m^−2^ (or above 35 kg m^−2^ in the presence of major comorbidity) when all non‐surgical measures have been tried without success, and should be prioritized for those with a BMI >50 kg m^−2^ and those with recent‐onset type 2 diabetes (T2D) with a BMI as low as 30 kg m^−2^ 3. Whilst it is highly effective, this option is only appropriate for certain people with obesity, and access is limited 3.

Anti‐obesity medications (AOMs) may be prescribed in primary care, as well as in specialist clinics, as an adjunct to lifestyle management. However, in recent history two AOMs have been withdrawn from the market due to safety concerns 9, 10, and the only remaining AOM that is widely prescribed (orlistat) has only modest efficacy, and side effects limit its use for many people with obesity 11, 12. In 2015, the European Medicines Agency (EMA) approved two additional AOMs, but neither is currently recommended by the National Institute for Health and Care Excellence (NICE). This article examines the challenges facing obesity management in the UK, in particular why AOMs have not been widely accepted in clinical practice. It considers whether the newer AOMs are likely to perform better, and how we can improve current clinical practice and future clinical trial design to identify those individuals most likely to benefit from AOMs and, thus, improve outcomes for people with obesity.

## The scale of the obesity epidemic in the UK

In 2015, approximately one‐quarter of adults in the UK had a BMI ≥30 kg m^−2^, which classes them as having obesity 13, 14, 15, 16. The prevalence of severe obesity (BMI ≥40 kg m^−2^) in 2015 was approximately 3% (England and Scotland) 15, 17. Obesity is associated with almost 200 metabolic, mechanical and mental comorbidities 18, many of which increase in prevalence as BMI rises 19. The risk of T2D, for example, rises exponentially with increasing BMI 20, which, in turn, increases the risk of cardiovascular (CV), microvascular and other complications 21. In 2012, the annual economic impact of obesity in the UK was £73 billion (3.0% gross domestic product [GDP]) and ranked second behind smoking (£90 billion; 3.6% GDP) 2. A simulated model predicts that by 2030 there could be an increase in the prevalence of obesity to up to 48% in men and up to 43% in women, equating to approximately 11 million more people with obesity in the UK 22. Combined medical costs for treatment of the complications of obesity are estimated to increase each year by up to £2 billion 22. The prevalence of severe obesity, which is associated with even greater healthcare costs, is also forecast to rise 13, 15, 16 (data not available for Northern Ireland); already over a million people are affected, and bariatric surgery is offered to less than 1% of this group annually 23. Projections largely indicate the BMI of people with overweight or obesity to be increasing over time 22, reflecting the chronic nature of the condition.

## Current guidelines for weight management in adults in the UK

**Figure 1:**
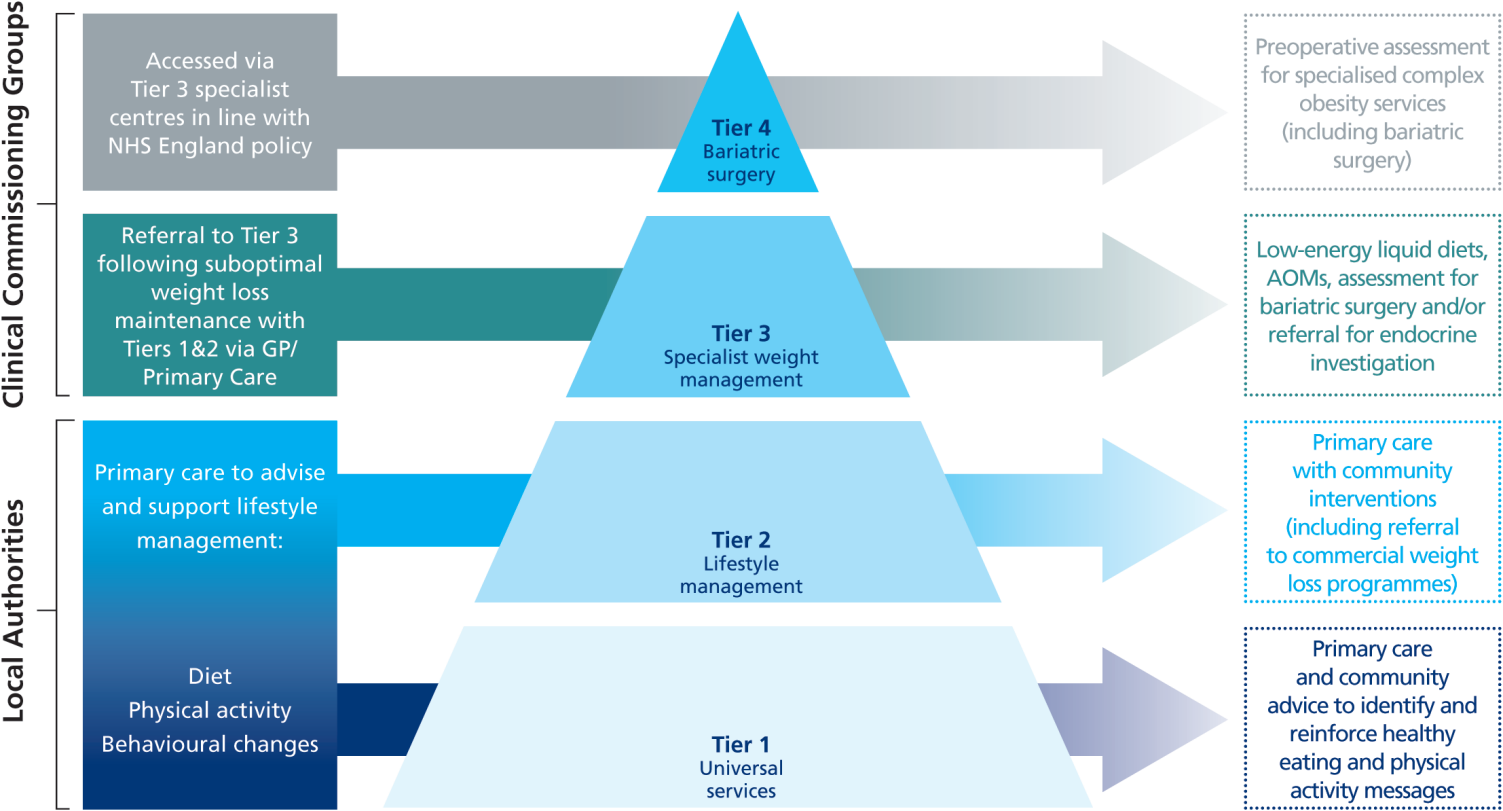
The 4‐tiered weight management approach recommended by the UK NHS commissioning board. AOM, anti‐obesity medication; GP, General Practitioner; NHS, National Health Service. Adapted from Commissioning guide 2014. Weight assessment and management (Tier 3). BOMSS, RCS, NICE.

In the UK, people with obesity primarily access weight loss interventions through primary care services 24, 25, 26 or by self‐referral to commercial slimming organizations. NICE and the Scottish Intercollegiate Guidelines Network (SIGN) provide guidance for multicomponent lifestyle weight management, based on the severity of obesity and the presence of weight‐related comorbidities 3, 5. Multicomponent lifestyle interventions (which include physical activity, dietary changes and behavioural components) should be the treatment of choice. AOMs should be considered on an individual case basis, where target weight has not been achieved through lifestyle interventions. In the UK, the NHS Commissioning Board recommends a 4‐tiered weight management approach (Fig. 1) 4.

### Tier 2 services in the UK

Commercial lifestyle weight management programmes, such as Rosemary Conley, Slimming World or Weight Watchers, demonstrate proven effectiveness at 12 months 27 and should help people with overweight or obesity to assess their weight and set realistic goals for weight loss, and support them in achieving it through long‐term, steady weight reductions using a multicomponent approach 28. In 2013, approximately 69 000 adults in UK were referred to Weight Watchers and Slimming World under the NHS schemes 4. Commercial programmes have been shown to be effective in the short term, with a 12‐week programme achieving approximately 5% body‐weight loss (4.8 kg); however, drop‐out rates are high 29. In the longer‐term (12 months), commercial programmes appear to be more effective than standard of care (i.e. weight loss advice from a general practitioner [GP]), with participants losing twice as much weight on the commercial programme 27. Despite the success of such programmes, for many, there is significant weight regain beyond the end of the programme 30. For people with a BMI ≥35 kg m^−2^ with additional risk factors, or >40 kg m^−2^ who are unable to achieve lasting weight loss through Tier 2 services, referral to Tier 3 weight management services is recommended to provide more intensive support, pharmacotherapy (if appropriate) and consideration for subsequent surgical referral 3.

### Tier 3 services in the UK

Several programmes have shown that Tier 3 services can be implemented successfully in primary care and that they may be more cost‐effective than Tier 3 services based in secondary care 24. The Scottish Government commissioned three phases of ‘The Counterweight Programme’, evaluated over 4 years (2001–2004) 31. Interventions delivered by practice nurses achieved an average weight loss of 3.0 kg for patients attending at 12 months and 2.3 kg for patients attending at 24 months 31. Over 30% of attendees maintained a weight loss of ≥5% at 12 months and at 24 months 31. This study demonstrated that, with the appropriate training and support, clinically significant weight loss can be achieved and maintained in primary care 31. The Rotherham Institute for Obesity (RIO), one of the first GP‐led services delivered in primary care, was initiated in 2010 and funded by Public Health Rotherham 26. It offered multidisciplinary services in primary care for people with a BMI ≥30 kg m^−2^ with additional risk factors, or >40 kg m^−2^ not achieving NHS Rotherham weight loss targets with Tier 2 support services 32. During the 2‐year period from 2010 to 2011, 66% of people completing the RIO programme met or exceeded weight loss targets 26. Of these successes, 72% of participants lost >5% of initial body weight and 19% of participants lost >10% 26. In 2017, Public Health Rotherham decommissioned RIO after 8 years 33. The Fakenham Weight Management Service (FWMS) was modelled on RIO and offered a 1‐year primary care programme to support weight loss through the implementation of sustainable lifestyle changes 24. The service (which reported on participants enrolled from August 2011 to August 2012) was evaluated using the National Obesity Observatory Standard Evaluation Framework (NOO SEF) and 117 of the 170 participants completed the programme 24. Of the ‘completers’, 72.6% had lost ≥5% of their initial body weight 24. In Liverpool, the Aintree Liverpool Obesity Support Service (LOSS) programme provides community‐based, multidisciplinary support for people with severe and complex obesity (average BMI 45.6 kg m^−2^) in a community setting 34. During the 4‐year period from 2009 to 2013, 2472 people were referred to the service; a retrospective follow‐up of 2315 appropriate referrals showed that, of those who engaged with the programme, over one‐third attending for longer than 6 months achieved a weight loss of more than 5% 34. This study also highlighted the difficulty in maintaining engagement, especially in younger individuals from areas of high socioeconomic deprivation 34. Despite the successes, there is evidence to suggest that Tier 3 services are often poorly implemented or lacking in some areas of the UK, with substantial regional variation 8, 35.

## Challenges associated with long‐term maintenance of weight loss

There are several reasons why a large proportion of people with obesity are unable to achieve long‐lasting weight loss. Following weight loss, the body compensates through several counter‐regulatory mechanisms that involve peripheral and central modulators of appetite and energy expenditure 36. Circulating concentrations of leptin, glucagon‐like peptide‐1, peptide‐YY and amylin decrease, impairing satiety, whereas plasma ghrelin levels increase, driving hunger 37, 38, 39, 40, 41, 42. In addition, weight loss is associated with slowing of metabolic rate that contributes to weight regain and progresses over time 7. Furthermore, it is difficult to maintain weight loss in the presence of strong environmental pressures that contributed to weight gain in the first place, e.g., easily accessible fast food outlets, or lack of cycling infrastructure 43.

## Anti‐obesity medications

The increasing prevalence of obesity in the UK and the understanding that many individuals may be unable to achieve and maintain weight loss with lifestyle interventions alone mean that the addition of newer AOMs to obesity management has become a more urgent priority. Orlistat (usual dose 120 mg three times daily [TDS] with meals) is currently the only AOM approved for long‐term use in the UK and recommended by NICE 44 (Table 1). Orlistat inhibits pancreatic lipase and reduces fat absorption in the gut 44. Orlistat should be stopped after 12 weeks if an individual has been unable to lose ≥5% of their body weight from start of treatment 44. Orlistat at a lower dose of 60 mg TDS is available over‐the‐counter in the UK and is indicated for weight loss in adults who are overweight (BMI ≥28 kg m^−2^), in combination with a mildly hypocaloric, lower‐fat diet 47. Sibutramine, a serotonin‐noradrenaline re‐uptake inhibitor, was available in the UK; however, post‐approval data showing a higher‐than‐anticipated incidence of CV adverse events (AEs) (e.g. myocardial infarction, stroke and cardiac arrest) in participants at high risk of CV disease led to withdrawal of its marketing authorization by the EMA in 2010 10. Rimonabant, a cannabinoid receptor 1 blocker, was available in the UK from 2006; however, post‐approval data showing serious psychiatric AEs led to its withdrawal in 2008 48.

**Table 1:** Overview of the AOMs currently approved for use in US/Europe 45, 46

AOM	Indication	MoA	Stopping rule	Approval status	NICE status	UK (NHS)
Orlistat 60 mg (OTC) or 120 mg (Rx)	Adjunct to a reduced‐calorie diet in obese BMI ≥30 kg m^−2^ overweight BMI ≥28 kg m^−2^ with comorbidities	Lipase inhibitor	Europe: Discontinue after 12 weeks if individual has been unable to lose ≥5% of initial BW	Europe: Approved (Rx & OTC) (1998/2007) US: Approved (Rx & OTC) (1999/2007)	Recommended	Available
PHEN/TPM ER 3.75 mg/23 mg 7.5 mg/46 mg 11.25 mg/69 mg 15 mg/92 mg	Adjunct to diet and physical activity for chronic weight management in: obese BMI ≥30 kg m^−2^ and overweight BMI ≥27 kg m^−2^ with comorbidity	NA and DA reuptake inhibitor and glutamate RA	Discontinue if ≥5% BW loss is not achieved after 12 weeks on maximum dose	Europe: Rejected (2013) US: Approved (2012)	N/A	Not available
Lorcaserin 10 mg		Selective 5‐HT_2c_ RA	Discontinue if the individual has not lost ≥5% of baseline BW by week 12	Europe: Application withdrawn (2013) US: Approved (2012)	N/A	Not available
Naltrexone/bupropion 8.0 mg/90 mg		μ‐opioid RA/NA and DA reuptake inhibitor	Europe: Discontinue after 16 weeks if individual has not lost ≥5% of their initial BW US: Discontinue if individual has not lost ≥5% of their baseline BW after 12 weeks of treatment at the maintenance dosage	Europe: Approved (2015) US: Approved (2014)	Not recommended	Not available
Liraglutide 3.0 mg		GLP‐1RA	Europe: Discontinue if individual has not lost ≥5% initial BW loss after 12 weeks of treatment on the 3.0 mg day^−1^ dose US: Discontinue if the individual has not lost ≥4% of baseline BW 16 weeks after treatment initiation	Europe: Approved (2015) US: Approved (2014)	Not reviewed to date; review by NICE to be determined	Availability via the NHS yet to be determined

5‐HT, serotonin; AE, adverse event; AOM, anti‐obesity medication; BMI, body mass index; BW, body weight; DA, dopamine; EMA, European Medicines Agency; ER, extended release; FAD, final appraisal determination; GLP‐1RA, glucagon‐like peptide‐1 receptor agonist; MoA, mechanism of action; NA, noradrenaline; N/A, not applicable; NHS, National Health Service; NICE, National Institute for Health and Care Excellence; OTC, over‐the‐counter; PHEN, phentermine; RA, receptor antagonist; Rx, prescription; TPM, topiramate.

A greater understanding of energy homeostasis and central appetite regulation has led to the development and recent regulatory approval of four other AOMs (Table 1). Naltrexone/bupropion (8.0 mg/90 mg two tablets twice daily [BID]) and liraglutide (3.0 mg once daily [OD] by subcutaneous injection) are approved for the long‐term treatment of obesity in both Europe and the US; both are subjected to additional monitoring for AEs 49, 50. Although naltrexone/bupropion and liraglutide 3.0 mg are approved by the EMA, neither drug has been recommended by NICE. In July 2017, NICE issued a final appraisal determination for naltrexone/bupropion, which did not recommend its use in the UK due to uncertainty over long‐term effectiveness and value for the NHS 51. The other two AOMs have been approved for long‐term treatment by the US Food and Drug Administration (FDA), but are not approved in Europe; they are phentermine/topiramate extended release (ER) (3.75 mg/23 mg; 7.5 mg/46 mg; 11.25 mg/69 mg; 15 mg/92 mg OD) and lorcaserin (10 mg BID). In 2012, the Committee for Medicinal Products for Human Use (CHMP) concluded that the benefits of phentermine/topiramate did not outweigh its risks (due to concerns over the long‐term effects on the heart and blood vessels, long‐term psychiatric effects, cognitive effects, teratogenicity and also that it would not be strictly used for the intended people) and recommended that it be refused marketing authorization 52. In February 2013, the CHMP reviewed the data for lorcaserin and had some concerns regarding the risk of tumours with long‐term use (based on non‐clinical studies) and the potential risk of psychiatric disorders and valvulopathy 53. Three months later, the manufacturers of lorcaserin notified the CHMP that they wished to withdraw their application for marketing authorization 53.

## How AOMs have translated into clinical practice to date: the efficacy–effectiveness gap

Despite the availability of AOMs in the UK, evidence suggests that they have not lived up to the promise shown in clinical trial programmes. Orlistat and sibutramine (when it was available) appear to be much less effective in general practice than in randomized clinical trials 54, 55. A retrospective analysis of data from the UK Clinical Practice Research Datalink (CPRD) in 2014 (that identified 100 701 people receiving orlistat, 15 355 receiving sibutramine and 508 140 receiving no intervention) showed that in UK clinical practice, orlistat and sibutramine had early effects on weight loss, but that these were not sustained over a 3‐year period 11. Findings from the CPRD suggest that treatment courses of orlistat are generally longer in clinical trials than in clinical practice, with 28% of people in the UK receiving only a single prescription (indicating that they received little or no treatment) 11. Prescribing records in the UK indicate that 2 years after initiating orlistat therapy, the median proportion of follow‐up is only 11% compared with approximately 50% in clinical trials 11.

With any medicine, there is a gap between the efficacy of an intervention under ideal clinical trial conditions and the effectiveness when it reaches clinical practice 56. The efficacy–effectiveness gap arises due to biological and behavioural variations in the real‐world setting compared with a clinical trial setting. The extent of this gap is dependent on the external validity of the clinical trial 56. Important components that contribute to external validity include: trial setting, patient recruitment, baseline characteristics, differences between the trial protocol and routine clinical practice and AE reporting 56, 57.

Recruitment of individuals to randomized clinical trials is carefully controlled via inclusion and exclusion criteria, resulting in a relatively homogeneous population under evaluation. Treatment with the drug under evaluation is optimized so that efficacy tends to be high and susceptibility to AEs low 56. Common limitations in phase 3 trials in obesity are that they recruit a population of mainly middle‐aged, Caucasian women with few comorbidities 58. As someone who works in Tier 3 services, I have observed that some sub‐populations are under‐represented in trials of AOMs, e.g. older adults or individuals with BMI >50 kg m^−2^, T2D, obstructive sleep apnoea (OSA) or mobility issues. This means that the efficacy and safety findings from trials of AOMs may not be generalizable to a large proportion of the people who are seen in Tier 3 services or, indeed, to many other people with obesity in the UK 58. In 2010, this was the case with a post‐approval analysis from the Sibutramine Cardiovascular Outcomes Trial (SCOUT); data suggested an increased risk of stroke and myocardial infarction compared with placebo 59. The indications for approved AOMs include people with overweight (BMI ≥27 kg m^−2^) with comorbidities (Table 1), which means people receiving AOMs in clinical practice may differ significantly from those who participate in clinical trials.

Clinical trial infrastructure is such that participants receive regular contact with a multidisciplinary team, and have health‐related quality of life and symptoms of depression monitored at regular intervals. In an ideal world, care in UK clinical practice should aim to reflect the approach used in clinical trials. Whilst we have examples of where this is possible (e.g. RIO, FWMS, Aintree LOSS 24, 26, 34), there is substantial regional variation in the provision of dietary advice, education (on obesity risk factors, lifestyle factors, obesity‐related health risks and weight loss options) and psychological support. The transfer of public health services from Primary Care Trusts to Local Authorities in 2013 resulted in further disruption to services 8.

There has been a recurring pattern of early promise and late failure in the clinical development of AOMs 60, and most AOMs developed to date have either not received regulatory approval or have been withdrawn from the market due to unacceptable side effects 60. It is perhaps then no surprise to primary care practitioners that the UK currently has only one NICE‐recommended prescription AOM and that commissioning bodies are reluctant to invest (or may even be disinvesting) in specialist Tier 3 support services.

## Will the recently approved AOMs change weight management practice in the UK?

### Building on previous experience

By identifying reasons for the suboptimal translation of current AOMs to clinical practice, we are able to assess if the more recently approved AOMs will fare any better and what clinicians can do to facilitate improved outcomes for people with obesity. Based on a review of the phase 3 randomized clinical trials of naltrexone/bupropion, liraglutide 3.0 mg and orlistat 120 mg, the following factors may influence the efficacy–effectiveness gap in the UK.

#### *Individuals with obesity and comorbidities*

The phase 3 trials of naltrexone/bupropion and liraglutide 3.0 mg (Table 2) have gone some way to addressing the efficacy–effectiveness gap with regard to people with obesity and comorbidities by evaluating the efficacy of naltrexone/bupropion in people with obesity and T2D and of liraglutide 3.0 mg in people with obesity and prediabetes, T2D or OSA. However, a gap still exists between the disease stage of people taking part in randomized clinical trials and those in a Tier 3 clinical practice setting. Those in Tier 3 typically have severe obesity, have accumulated a large number of comorbidities 34 and found that Tier 2 support is not sufficient. The cost‐effectiveness models used by NICE are based on phase 3 data and a limited set of outcomes, so may not fully take into account the beneficial effects of weight loss in cases of people with severe obesity with multiple complications.

**Table 2:** Current clinical evidence for efficacy of AOMs in people with overweight or obesity

Clinical trial identifier	Intervention	Clinical trial population	Ineligibility	Study centres	Assessments	Statistical analysis	Completers
**Naltrexone/bupropion**							
COR‐I 58	Mild hypocaloric diet (500 kcal day^−1^ deficit) and exercise, plus randomization (1:1:1) to: NB32 NB16 Placebo for 56 weeks	Adults (18–65 years) with BMI 30–45 kg m^−2^ or 27–45 kg m^−2^ with hypertension and/or dyslipidaemia 1482 females 260 males	Obesity of known endocrine origin; T1D/T2D; cerebrovascular, cardiovascular, hepatic or renal disease; previous surgical or device intervention for obesity; loss/gain of 4 kg before randomization; pregnant and lactating women; history of seizures or serious psychiatric illness; bupropion or naltrexone treatment in previous 12 months; history of drug or alcohol use in previous 12 months	34 sites in the US Combination of academic and primary care centres	Screening, and every 4 weeks Compliance with diet and exercise instruction not obtained IWQoL‐Lite, FCI, COEQ used at baseline and at weeks 8, 16, 28 and 56 Depressive symptoms assessed using IDS‐SR Drug compliance measured at every visit Non‐compliant participants (<70% compliance rate) received counselling and re‐education If non‐compliance for 2 consecutive months or for 15 consecutive days they were considered for discontinuation from study	The primary analysis population included all randomized participants with a baseline weight measurement and a post‐baseline weight measurement while on study drug Missing data were imputed by LOCF	870 (50%) completed 56 weeks of NB treatment Discontinuations in the NB groups due to AEs Mostly by weeks 4 and 8 Discontinuation due to drug non‐compliance NB32: 2.9% NB16: 1.4% Placebo: 2.6%
COR‐II 61	Hypocaloric (500 kcal day^−1^ deficit) diet and increased physical activity, plus randomization (2:1) to: NB32 Placebo for 56 weeks Participants with suboptimal response re‐randomized weeks 28–44 (1:1) to: NB32 NB48 (note this dose is not licensed)	Adults (18–65 years) with BMI 30–45 kg m^−2^ or 27–45 kg m^−2^ with controlled hypertension and/or dyslipidaemia	Diabetes; significant vascular, hepatic or renal disease; weight change of >4 kg within 3 months prior to randomization; history of seizures or serious psychiatric illness	36 US private/institutional practices	Study visits occurred at baseline and every 4 weeks Weight and vital signs measured at each visit At baseline, 12, 24, 36 and 48 weeks, participants received instructions to follow a hypocaloric diet and increase physical activity, and behavioural modification advice IWQoL‐Lite, COEQ used Depressive symptoms assessed using IDS‐SR	Efficacy analyses were performed on a prespecified mITT analysis population comprising all randomized participants with a baseline weight measurement and a post‐baseline weight measurement while on study drug Missing data were imputed by LOCF	805 (54%) completed 56 weeks of NB32 treatment Discontinuation in the NB groups due to AEs, and in the placebo group due to insufficient weight loss or withdrawal of consent Discontinuations occurred most frequently in the first 8 weeks
COR diabetes 62	Hypocaloric (500 kcal day^−1^ deficit) diet and increased physical activity, plus randomization (2:1) to: NB32 Placebo	Adults (18–70 years) with BMI ≥27 to ≤45 kg m^−2^ with HbA_1c_ 7–10%, FPG <270 mg/dL (<15.0 mmol/L^a^) and SBP/DBP <145/<95 mmHg 1625 participants screened	T1D, obesity of unknown endocrine origin, serious medical conditions (including, but not limited to ongoing renal/hepatic insufficiency, Class III/IV CHF, MI, angina pectoris, lifetime history of stroke), severe microvascular/macrovascular complications of diabetes, serious psychiatric illness	53 US sites	At week 2, participants received a telephone call to assess study medication compliance Participant assessments were undertaken at screening, baseline, and every 4 weeks thereafter Participants received dietary counselling, advice on behavioural modification and on increasing physical activity Participants were instructed to monitor their blood glucose twice daily for the first 4 weeks	Primary efficacy analyses were performed using the mITT population comprising all randomized participants with a baseline measurement and one or more post‐baseline measurements of body weight while on the study drug Missing data were imputed by LOCF	175 (52.2%) in the NB group and 100 (58.8%) in the placebo group completed the 56‐week treatment period Over the 56‐week trial, 47% of participants in the NB group discontinued the study drug compared with 41.2% in the placebo group A greater percentage of participants receiving NB withdrew due to AEs *versus* placebo (29.3 *versus* 15.3%) A greater proportion of people receiving placebo were lost to follow‐up (8.8 *versus* 6.3%), withdrew consent (8.8 *versus* 6.3%) or withdrew due to self‐perceived insufficient weight loss (3.5 *versus* 1.5%)
**Liraglutide 3.0 mg**							
SCALE obesity and prediabetes 63, 64	Hypocaloric diet and increased physical activity, plus randomization (2:1) to: Liraglutide 3.0 mg Placebo After 56 weeks, participants in the liraglutide group without prediabetes at screening were randomized (1:1) for 12 weeks to: Continue liraglutide 3.0 mg Placebo	Adults with or without prediabetes, with a BMI ≥30 kg m^−2^, or ≥ 27 kg m^−2^ if they had untreated dyslipidaemia or hypertension	Diabetes, use of medications that cause clinically significant weight gain/loss, previous bariatric surgery, history of pancreatitis, history of severe psychiatric disorders, family or personal history of multiple endocrine neoplasia type 2 or familial medullary thyroid carcinoma	191 sites in 27 countries in Europe, North America, South America, Asia, Africa and Australia	Screening Every 2 weeks until week 8; then every 4 weeks until week 44; then again at weeks 50, 56, 58, 60, 64, 68 and 70 Standardized lifestyle modification counselling approximately monthly Individuals who withdrew early returned at week 56 for measurement of their weight and recording of AEs HRQoL measured using IWQoL‐Lite and SF‐36 AEs occurring during trial period, with onset on or after first day of treatment and up to 14 days after last day of treatment	Full analysis set included all participants randomized who received ≥1 dose of study drug and ≥ 1 assessment after baseline Missing data were imputed by LOCF	1789 (71.9%) participants with or without prediabetes completed 56 weeks of treatment with liraglutide 3.0 mg 791 (53%) of participants with prediabetes completed 160 weeks of treatment with liraglutide 3.0 mg 65 More participants in the liraglutide group withdrew from the trial due to AEs More participants in the placebo group withdrew from the trial owing to ineffective therapy or withdrew their consent
SCALE diabetes 65	Hypocaloric (500 kcal day^−1^ deficit) diet and increased physical activity, plus randomization (2:1:1) to: Liraglutide 3.0 mg Liraglutide 1.8 mg (not approved for weight management) Placebo	BMI ≥27 kg m^−2^ and T2D	Treatment with any GLP‐1RA or DPP‐4 inhibitor or insulin with in the last 3 months; treatment with any hypoglycaemic agent(s) other than metformin, sulphonylurea and glitazone in the last 3 months; untreated/ uncontrolled hypertension or hypo/hyperthyroidism; history of serious psychiatric disorders	126 sites in nine countries (France, Germany, Israel, South Africa, Spain, Sweden, Turkey, UK [England and Scotland only], US)	Body weight measured at every visit to Week 68 Safety and tolerability assessments recorded at every visit HRQoL measured using IWQoL‐Lite and DTSQ status version	Full analysis set included all participants exposed to ≥1 treatment dose with ≥1 post‐baseline efficacy assessment The three coprimary weight endpoints were analysed using a multiple imputation approach; all other efficacy variables were analysed using LOCF	324 (76.6%) in the liraglutide 3.0 mg group, and 140 (66.0%) in the placebo group completed the 56‐week treatment period
SCALE maintenance 66	Individuals who lost ≥5% initial body weight during the 4–12‐week run‐in (on a 1200–1400 kcal day^−1^ diet) were prescribed a 500 kcal day^−1^ deficit diet and regular exercise (150 min week^−1^ of brisk walking recommended) randomized (1:1) to: Liraglutide 3.0 mg Placebo	Subjects maintaining prior weight loss achieved with a low‐calorie diet and with stable body weight and BMI ≥30 kg m^−2^ or > 27 kg m^−2^ with comorbidities of treated or untreated dyslipidaemia and/or treated or untreated hypertension	Diabetes; FPG ≥7 mmol L^−1^ at run‐in, treatment with GLP‐1RA or medications causing significant weight gain/loss, bariatric Surgery, history of idiopathic acute or chronic pancreatitis, history of severe psychiatric disorders clinically significant active CVD	26 sites in the US and 10 sites in Canada	Medical monitoring at weeks 0, 1, 2, 3 and 4 and Weeks 6, 10, 14, 18, 22, 26, 30, 34, 38, 42, 46 and 52, for a total of 17 visits over 56 weeks Mental health assessed by the CSSRS and the PHQ‐9	Full analysis set included all randomized individuals exposed to trial drug with ≥1 post‐randomization weight assessment Missing data were imputed by LOCF	159 (75%) in the liraglutide group and 146 (69.5%) in the placebo group completed the 56‐week treatment period
SCALE Sleep Apnoea 67	Hypocaloric (500 kcal day^−1^ deficit) diet and increased physical activity, plus randomization (1:1) to: Liraglutide 3.0 mg Placebo	Subjects with BMI ≥30 kg m^−2^ and with a stable body weight for the prior 3 months, had a diagnosis of moderate or severe OSA, and were unwilling/unable to use CPAP (or other positive airway pressure) treatment within the 4 weeks prior to screening	Central sleep apnoea, diabetes or HbA_1c_ ≥ 6.5%, significant craniofacial abnormalities that may be causing OSA, previous surgical treatment for obesity	40 sites in the US and Canada	Body weight parameters and vitals assessed every visit from Week 0 to 32 Polysomnographic assessments at Weeks −1, 12 and 32 After randomization, glycaemic parameters, fasting lipids and cardiovascular biomarkers were assessed at Weeks 0, 12 and 32; FPG was also assessed at Weeks 4, 8, 20 and 28 QoL assessed with FOSQ and SF‐36 at Weeks 0, 12 and 32 Mental health assessed by the CSSRS and the PHQ‐9	Full analysis set included all randomized participants LOCF was pre‐specified for primary and secondary analyses	134 (74%) in the liraglutide group and 142 (79%) in the placebo group completed the trial More participants in the liraglutide group withdrew due to AEs Gastrointestinal AEs most commonly led to withdrawal in liraglutide group
**Orlistat**							
XENDOS 55	Hypocaloric (800 kcal day^−1^ deficit) diet and increased physical activity, plus randomization (1:1) to: Orlistat 120 mg Placebo Every 6 months, energy intake was assessed to account for weight loss achieved. Participants received dietary counselling every 2 weeks (first 6 months) and monthly thereafter	BMI ≥30 kg m^−2^ and without diabetes (but may have impaired glucose tolerance)	Diabetes; ongoing and active cardiovascular or gastrointestinal disease	22 medical centres in Sweden	75 g OGTT was performed at baseline and then at every 6 months After the first 6 months, participants with a diabetic OGTT underwent a repeat OGTT within 4 weeks Individuals diagnosed with T2D remained in the study and had fasting whole blood glucose levels measured at 6‐month intervals Body weight recorded at every study visit (every 3 months) Waist circumference assessed at baseline, Months 3 and 6, and every 6 months thereafter Laboratory parameters assessed every 6 months	ITT population included all randomized participants who received ≥1 dose of study drug and had ≥1 follow‐up assessment Descriptive statistics for change in body weight and categorical weight changes used LOCF data	850 (52%) in the orlistat group and 564 (34%) in the placebo group completed 4 years of treatment The most common causes of withdrawal in both groups were refusal of treatment and insufficient therapy response Comparable drug compliance in both groups: Orlistat: 93.3% Placebo: 92.8%

a Conversion of mg/dL to mmol L^−1^ using a factor of 18.

AE, adverse event; AOM, anti‐obesity medication; BMI, body mass index; CHF, congestive heart failure; COEQ, Control of Eating Questionnaire; COR, CONTRAVE Obesity Research; CPAP, continuous positive airway pressure therapy; CSSRS, Columbia Suicide Severity Rating Scale; CVD, cardiovascular disease; DBP, diastolic blood pressure; DPP‐4, dipeptidyl peptidase‐4; DTSQ, Diabetes Treatment Satisfaction Questionnaire; FCI, Functional Comorbidity Index; FOSQ, Functional Outcomes in Sleep Questionnaire; FPG, fasting plasma glucose; GLP‐1RA, glucagon‐like peptide‐1 receptor agonist; HbA_1c_, glycated haemoglobin; HRQoL, health‐related quality of life; IDS‐SR, Inventory of Depressive Symptomatology Self Report; ITT, intention to treat; IWQoL‐Lite, Short Form of Impact of Weight on Quality of Life‐Lite; LOCF, last observation carried forward; MI, myocardial infarction; mITT, modified intention to treat; NB16, sustained‐release naltrexone 16 mg/sustained‐release bupropion 360 mg fixed‐dose tablet; NB32, sustained‐release naltrexone 32 mg/sustained‐release bupropion 360 mg fixed‐dose tablet; NB48, sustained‐release naltrexone 48 mg/sustained‐release bupropion 360 mg fixed‐dose tablet; OGTT, oral glucose tolerance test; OSA, obstructive sleep apnoea; PHQ‐9, Patient Health Questionnaire‐9; QoL, quality of life; SBP, systolic blood pressure; SCALE, Satiety and Clinical Adiposity – Liraglutide Evidence (in individuals with and without diabetes); SF‐36, Short Form Health Survey 36; T1D, type 1 diabetes; T2D, Type 2 diabetes; XENDOS, XENical in the prevention of diabetes in obese participants.

#### *Responders* versus *non‐responders*

The prescribing rules for AOMs require an evaluation of response after 12 weeks at the intended clinical dose (after any titration period). Individuals who have not lost ≥5% of baseline weight after 12 weeks of intended clinical dose are considered ‘non‐responders’ and the treatment should be stopped (according to the EMA stopping rule), whereas individuals achieving this clinically meaningful weight loss target after 12 weeks at intended clinical dose are considered ‘responders’. For example, in the Satiety and Clinical Adiposity – Liraglutide Evidence (SCALE) in individuals with and without diabetes Obesity and Prediabetes trial, the proportion of all subjects (responders and non‐responders) achieving a ≥5% weight loss at week 56 with liraglutide 3.0 mg was 63.2% (*versus* 27.1% with placebo) but was 88.2% for responders (*versus* 36.9% for non‐responders) 68. This type of analysis may better describe the likely real‐world effectiveness, assuming the stopping rule is followed.

#### *Reduction of risk factors*

In the phase 3 trials of naltrexone/bupropion, liraglutide 3.0 mg and orlistat 120 mg (Table 2) there was evidence of the broader clinical benefit of AOMs. XENDOS (XENical in the prevention of Diabetes in Obese Subjects) study data show that 4 years of orlistat treatment confers a statistically significant improvement in waist circumference, diastolic and systolic blood pressure, fasting plasma glucose (FPG) and total cholesterol *versus* placebo 55. COR‐I (CONTRAVE Obesity Research I) data show that 56 weeks of naltrexone/bupropion treatment confers a statistically significant improvement in FPG levels, fasting insulin and insulin resistance *versus* placebo 58. SCALE Obesity and Prediabetes study data show that responders completing 56 weeks of treatment with liraglutide 3.0 mg experience a greater reduction in glycated haemoglobin (HbA_1c_), FPG, systolic and diastolic blood pressure, and that all measures of fasting lipids, high‐sensitivity C‐reactive protein, plasminogen activator inhibitor‐1 and adiponectin were improved *versus* placebo 63. Furthermore, an extension of the trial in people with obesity and prediabetes shows that, while on treatment, more individuals in the liraglutide 3.0 mg group (970/1472; 66%) had regressed from prediabetes to normoglycaemia by week 160 than had individuals in the placebo group (268/738; 36%) 64. SCALE Diabetes study data show that 56 weeks of liraglutide 3.0 mg treatment significantly improved HbA_1c_ and FPG levels; the proportion of participants achieving American Diabetes Association (ADA) and American Association of Clinical Endocrinologists (AACE) HbA_1c_ target levels was significantly greater than with placebo in people with obesity and T2D. In addition, systolic blood pressure, lipid profile (total cholesterol, very‐low‐density lipoprotein, high‐density lipoprotein and triglycerides) and some CV biomarkers (plasminogen activator inhibitor 1 and urinary albumin to creatinine ratio) were also improved *versus* placebo 65. SCALE Sleep Apnoea study data show that participants in the liraglutide 3.0 mg group had a significantly greater reduction in the number of apnoea–hypopnoea events/hour at week 32, compared with the placebo group 67.

As described above, there are a number of factors that may affect the translation of recently approved AOMs into UK clinical practice. Yet, we have also seen that there are a multitude of clinical benefits that could be gained by people with obesity in addition to weight loss. With this in mind, what can we do moving forward to reduce the efficacy–effectiveness gap in the UK and, ultimately, to improve patient outcomes?

## Improving outcomes for people with obesity in the UK

Given the burden that obesity places on the lives and health of the people it affects, and the strain that the disease and its complications place on our healthcare system, it is important that appropriate interventions are available to help treat those who are overweight or have obesity. With the prevalence of obesity predicted to continue to rise 22, there is also a need to make environmental changes to lessen the future epidemic. Key aspects that could reduce the efficacy–effectiveness gap include applying best practice to ensure equitable access to all four tiers of obesity care (from prevention through to bariatric surgery) nationwide (particularly ensuring additional support for those with more severe and complicated obesity in Tier 3 services), and undertaking real‐world effectiveness trials to help optimize the use of currently available AOMs. This may offer cost‐effective implementation within the NHS, particularly for individuals who may benefit, thereby closing the efficacy–effectiveness gap.

### Driving improvements in the provision of Tier 3 support services

There are exemplary cases where Tier 3 services and commissioning guideline recommendations have been implemented, but these need to be applied consistently throughout the UK to address a number of key barriers. These include the belief (by both people with obesity and commissioners) that medical therapy is not effective, constraints on medical workloads, a lack of trained staff, financial constraints and the challenges of NICE evaluation 8. Moreover, some commissioners believe that Tier 3 services are simply a bridge to bariatric surgery and that successfully achieving weight loss with AOMs (without the need for bariatric surgery) is unlikely 8. More needs to be done to improve the efficiency of Tier 3 services, e.g. through partnerships with other NHS schemes or commercial organizations and the use of health trainers and group sessions. These services should, however, be tailored to the complex needs of people with obesity who have had multiple failures to maintain weight loss with Tier 2 support. The NOO SEF recommends Tier 3 services collect data on diet, physical activity and quality of life in addition to demographic data 69, but this requires dedicated administrators and a large database. The NOO SEF does not recognize important changes such as HbA_1c_ in people with T2D 69, yet additional clinical outcomes, including psychological outcomes and quality of life, should be reported to reflect the clinical and economic gains over and above weight loss that are provided by AOMs. These outcomes can be built into clinical systems (as has been done e.g. with the National Diabetes Audit – NHS Digital 70) and used to drive improvements in care forward. Partnerships with academic centres to improve robust data collection would help teams demonstrate the value of Tier 3 services to the NHS 8.

### Support for people with significant comorbidity

The medical costs for people with T2D are greater than for those without, and increase with every additional kg m^−2^ of BMI 71. For people with severe obesity this difference amounts to thousands of pounds per year 70. In 2016, the NHS began to roll out a Diabetes Prevention Programme (NHS DPP) 72. By 2020, up to 100 000 places are to be made available for people at high risk of T2D (just 1% of the 10 million at risk) to receive personalized support to reduce their risk, including education on healthy eating and lifestyle, help to lose weight and bespoke physical exercise programmes 72. In 2017, a translational research study of 166 people referred to Weight Watchers via the NHS DPP showed that after 1 year the mean weight reduction was 3.2 kg m^−2^, and 38% individuals returned to normoglycaemia 73. Health Survey for England (HSE) data suggest that some people with obesity and non‐diabetic hyperglycaemia (HbA_1c_: 42.0–47.0 mmol mol^−1^ or FPG: 5.5–6.9 mmol L^−1^) would be eligible to access NHS DPP support services (in addition to Tier 3 services for those with BMI >40 kg m^−2^) 74. If this were successful in preventing people with obesity from developing T2D, it may help to reduce the burden on the healthcare economy over the longer term.

### Optimizing the use of AOMs

It is clear that some gaps exist in terms of efficacy in clinical trials *versus* effectiveness in UK clinical practice, and there are also concerns about safety, based on previously withdrawn AOMs. However, AOMs offer a number of clinical benefits to people with obesity (including reductions in waist circumference, diastolic and systolic blood pressure, FPG and total cholesterol) 55, 58, 61, 62, 63, 65, 66, 67. Based on evidence from the CPRD regarding orlistat 120 mg, the shorter treatment duration that was seen in clinical practice compared with clinical trials may contribute to the efficacy–effectiveness gap 11. Four AOMs have demonstrated greater weight loss than lifestyle interventions in clinical trials, and importantly, aid in the long‐term maintenance of weight loss afterwards 55, 58, 61, 62, 63, 65, 66, 67. Liraglutide 3.0 mg has published clinical trial data supporting efficacy for people with obesity and prediabetes up to 3 years 64. Use of these AOMs also reduces obesity‐associated risk factors, including onset of T2D, dyslipidaemia and hypertension 55, 58, 61, 62, 63, 65, 66, 67. Furthermore, the more encompassing design of clinical trial programmes such as SCALE suggest that the more recently approved AOMs may go some way to addressing the efficacy–effectiveness gap.

Previous experience might give cause for concern about the safety of AOMs once they enter clinical practice; however, a *post hoc* analysis of the SCOUT trial data suggested that the excess of AEs with sibutramine occurred in non‐responders who continued the drug to the end of the clinical trial period 75. In clinical practice, the Summary of Product Characteristics for each of the current EMA‐approved AOMs include the stopping rule (Table 1), meaning that non‐responders should not continue taking the drug. Consideration should be given to how best these medications can be employed to optimize outcomes for people with obesity whilst bearing in mind cost‐effectiveness, and key to this is to identify populations of people with obesity who will respond well to therapy.

Moving forward, robust real‐world evidence studies are needed to accurately assess the effectiveness of targeted approaches to weight management. Using an example from T2D, the Diabetes REmission Clinical Trial (DiRECT) has successfully demonstrated that the benefits of a structured weight management programme can be achieved in a real‐world primary care setting at 12 months, with longer term data awaited 76. For obesity, a phase 4 clinical trial (STRIVE; NCT03036800) is currently underway to assess the weight loss (≥15% at 52 weeks; primary outcome), improvement in obesity‐related comorbidities, long‐term cost‐effectiveness and budget impact of specialist Tier 3 care and targeted AOM use in 400 people with severe and complex obesity in the UK.

## The future landscape of pharmacological weight loss interventions in the UK

Addressing the key aspects above, in order to reduce the efficacy–effectiveness gap of AOMs in the UK, will be challenging. Naltrexone/bupropion and liraglutide 3.0 mg have gone some way to changing the perception of, and building confidence in, the use of AOMs. Several promising new AOMs currently under evaluation (semaglutide [NCT02453711], exenatide extended‐release [NCT02794402] and the dual GLP‐1/glucagon receptor (GCGR) agonist SAR425899 [NCT03414736]) are predicted to show even greater efficacy than currently approved AOMs 77. It is possible that new AOMs may lead to improved clinical outcomes and, ultimately, to a further step change in the perception of AOMs and acceptance of their use as cost‐effective adjuncts to lifestyle intervention for some people with obesity. However, while changing perceptions and increasing acceptance of AOMs is an important step, other challenges remain.

Without a robust economic analysis that demonstrates cost‐effectiveness and impact on body‐weight‐related disease burden, currently approved and new AOMs will not fit within the NICE model. If we can provide appropriate and long‐term support for people with overweight and obesity in the UK (relative to their BMI and degree of comorbidity), broaden the eligibility criteria for clinical trials so that the data generated more accurately represent the people we treat on a daily basis, and identify subgroups of people with obesity most likely to benefit from AOMs, we can start to close the efficacy–effectiveness gap and to change the treatment landscape in the UK.

## Conflict of Interest Statement

Professor John Wilding is a Principal Investigator in the STRIVE Study (NCT03036800) and has undertaken advisory and educational work on obesity and/or diabetes for Astellas, AstraZeneca, Boehringer Ingelheim, Janssen, Lilly, Novo Nordisk, Orexigen and Takeda.

## References

[1] OECD . Organisation for Economic Co‐operation and Development Health Statistics: Obesity update, 2017 URL http://www.oecd.org/els/health-systems/Obesity-Update-2017.pdf (accessed 20 December 2017).

[2] McKinsey Global Institute . Overcoming Obesity: An Initial Economic Crisis. McKinsey, 2014.

[3] National Institute for Health and Care Excellence (NICE) . Obesity: identification, assessment and management (CG189), 2014 URL https://www.nice.org.uk/guidance/cg189/resources/obesity-identification-assessment-and-management-pdf-35109821097925 (accessed 20 December 2017). 36719951

[4] National Institute for Health and Care Excellence (NICE) . Weight management: lifestyle services for overweight or obese adults (PH53), 2014 URL https://www.nice.org.uk/guidance/ph53/resources/weight-management-lifestyle-services-for-overweight-or-obese-adults-1996416726469 (accessed 20 December 2017).

[5] Scottish Intercollegiate Guidelines Network (SIGN) . Management of obesity: a national clinical guideline, 2010 URL http://www.sign.ac.uk/assets/sign115.pdf (accessed 20 December 2017).

[6] Jensen MD , Ryan DH , Apovian CM *et al* 2013 AHA/ACC/TOS guideline for the management of overweight and obesity in adults: a report of the American College of Cardiology/American Heart Association task force on practice guidelines and the Obesity Society. Circulation 2014; 129: S102–S138. 24222017 10.1161/01.cir.0000437739.71477.ee PMC5819889

[7] Mann T , Tomiyama AJ , Westling E *et al* Medicare's search for effective obesity treatments: diets are not the answer. Am Psychol 2007; 62: 220–233. 17469900 10.1037/0003-066X.62.3.220

[8] Hughes CA . The rewards and challenges of setting up a tier 3 adult weight management service in primary care. Br J Obes 2015; 1: 25–31.

[9] EMA . Public statement on Acomplia^®^ (rimonabant) 2009 URL http://www.ema.europa.eu/docs/en_GB/document_library/Public_statement/2009/11/WC500012189.pdf (accessed 20 December 2017).

[10] EMA . Questions and answers on the suspension of medicines containing sibutramine, 2010 URL http://www.ema.europa.eu/docs/en_GB/document_library/Referrals_document/Sibutramine_107/WC500094238.pdf (accessed 20 December 2017).

[11] Douglas IJ , Bhaskaran K , Batterham RL , Smeeth L . The effectiveness of pharmaceutical interventions for obesity: weight loss with orlistat and sibutramine in a United Kingdom population‐based cohort. Br J Clin Pharmacol 2015; 79: 1020–1027. 25641659 10.1111/bcp.12578 PMC4456134

[12] Maahs D , de Serna DG , Kolotkin RL *et al* Randomized, double‐blind, placebo‐controlled trial of orlistat for weight loss in adolescents. Endocr Pract 2006; 12: 18–28. 16524859 10.4158/EP.12.1.18

[13] NHS . Health Survey for England (NHS Digital), 2015 URL http://www.content.digital.nhs.uk/catalogue/PUB22610/HSE2015-Sum-bklt.pdf (accessed 20 December 2017).

[14] Health Survey Northern Ireland . 2015/2016 [WWW document]. URL https://www.health-ni.gov.uk/sites/default/files/publications/health/hsni-first-results-15-16.pdf (accessed 20 December 2017).

[15] Scottish Health Survey . 2015 [WWW document]. URL http://www.gov.scot/Publications/2016/09/2764 (accessed 20 December 2017).

[16] Welsh Health Survey . 2015 [WWW document]. URL http://gov.wales/statistics-and-research/welsh-health-survey/?lang=en (accessed 20 December 2017).

[17] Baker C . Obesity statistics: briefing paper. House of Commons Library 2017; 3336: 1–20.

[18] Yuen MS , Lui DT , Kaplan LM *et al* A systematic review and evaluation of current evidence reveals 195 obesity‐associated disorders (OBAD). Obesity Week; New Orleans, Louisiana, US (Poster T‐P‐3166) 2016.

[19] Must A , Spadano J , Coakley EH *et al* The disease burden associated with overweight and obesity. JAMA 1999; 282: 1523–1529. 10546691 10.1001/jama.282.16.1523

[20] Abdullah A , Peeters A , de Courten M , Stoelwinder J . The magnitude of association between overweight and obesity and the risk of diabetes: a meta‐analysis of prospective cohort studies. Diabetes Res Clin Pract 2010; 89: 309–319. 20493574 10.1016/j.diabres.2010.04.012

[21] Marathe PH , Gao HX , Close KL . American diabetes association standards of medical Care in Diabetes 2017. J Diabetes 2017; 9: 320–324. 28070960 10.1111/1753-0407.12524

[22] Wang YC , McPherson K , Marsh T , Gortmaker SL , Brown M . Health and economic burden of the projected obesity trends in the USA and the UK. Lancet 2011; 378: 815–825. 21872750 10.1016/S0140-6736(11)60814-3

[23] (BOMSS) BOaMSS . Patient access to bariatric surgery 2017. URL http://www.bomss.org.uk/wp-content/uploads/2017/03/RCS-and-BOMSS-Bariatric-report-2017.pdf (accessed 20 December 2017).

[24] Jennings A , Hughes CA , Kumaravel B *et al* Evaluation of a multidisciplinary tier 3 weight management service for adults with morbid obesity, or obesity and comorbidities, based in primary care. Clin Obes 2014; 4: 254–266. 25825858 10.1111/cob.12066 PMC4253319

[25] Morrison DS , Boyle S , Morrison C *et al* Evaluation of the first phase of a specialist weight management programme in the UK National Health Service: prospective cohort study. Public Health Nutr 2012; 15: 28–38. 21806868 10.1017/S1368980011001625

[26] Senior L , Carter D , Capehorn M . Service evaluation of the Rotherham Institute for Obesity and comparison of 2010 and 2011 data. Obes Facts 2013; 6: 49–230 (Poster 084).

[27] Ahern AL , Wheeler GM , Aveyard P *et al* Extended and standard duration weight‐loss programme referrals for adults in primary care (WRAP): a randomised controlled trial. Lancet 2017; 389: 2214–2225. 28478041 10.1016/S0140-6736(17)30647-5 PMC5459752

[28] National Institute for Health and Care Excellence (NICE) . Obesity: guidance on the prevention, identification, assessment and management of overweight and obesity in adults and children, 2006 URL https://www.nice.org.uk/guidance/cg189/evidence/obesity-update-appendix-p-6960327450 (accessed 20 December 2017).

[29] Dixon KJ , Shcherba S , Kipping RR . Weight loss from three commercial providers of NHS primary care slimming on referral in North Somerset: service evaluation. J Public Health (Oxf) 2012; 34: 555–561. 22611263 10.1093/pubmed/fds034

[30] Holzapfel C , Cresswell L , Ahern AL *et al* The challenge of a 2‐year follow‐up after intervention for weight loss in primary care. Int J Obes (Lond) 2014; 38: 806–811. 24030517 10.1038/ijo.2013.180 PMC4052429

[31] Counterweight Project Team . Evaluation of the counterweight Programme for obesity management in primary care: a starting point for continuous improvement. Br J Gen Pract 2008; 58: 548–554. 18682018 10.3399/bjgp08X319710 PMC2486382

[32] RIO . How can I be referred (Rotherham Institute for Obesity), 2017 URL http://www.rotherhaminstituteforobesity.co.uk/How-can-I-be-referred.html (accessed 20 December 2017).

[33] Upton M . Huge funding blow for Rotherham Insitute for obesity. Rotherham Advertiser 2017 (accessed 20 December 2017).

[34] Steele T , Narayanan RP , James M *et al* Evaluation of Aintree LOSS, a community‐based, multidisciplinary weight management service: outcomes and predictors of engagement. Clin Obes 2017; 7: 368–376. 28871633 10.1111/cob.12216

[35] National Institute for Health and Care Excellence (NICE) . Accessing NHS care and treatment recommended by NICE, 2014 URL https://www.nice.org.uk/corporate/ecd4/resources/accessing-nhs-care-and-treatment-recommended-by-nice-pdf-1124012418757 (accessed 20 December 2017).

[36] Lean ME , Malkova D . Altered gut and adipose tissue hormones in overweight and obese individuals: cause or consequence? Int J Obes (Lond) 2016; 40: 622–632. 26499438 10.1038/ijo.2015.220 PMC4827002

[37] Chearskul S , Delbridge E , Shulkes A , Proietto J , Kriketos A . Effect of weight loss and ketosis on postprandial cholecystokinin and free fatty acid concentrations. Am J Clin Nutr 2008; 87: 1238–1246. 18469245 10.1093/ajcn/87.5.1238

[38] Cummings DE , Weigle DS , Frayo RS *et al* Plasma ghrelin levels after diet‐induced weight loss or gastric bypass surgery. N Engl J Med 2002; 346: 1623–1630. 12023994 10.1056/NEJMoa012908

[39] Geldszus R , Mayr B , Horn R *et al* Serum leptin and weight reduction in female obesity. Eur J Endocrinol 1996; 135: 659–662. 9025708 10.1530/eje.0.1350659

[40] Keim NL , Stern JS , Havel PJ . Relation between circulating leptin concentrations and appetite during a prolonged, moderate energy deficit in women. Am J Clin Nutr 1998; 68: 794–801. 9771856 10.1093/ajcn/68.4.794

[41] Leibel RL , Hirsch J . Diminished energy requirements in reduced‐obese patients. Metabolism 1984; 33: 164–170. 6694559 10.1016/0026-0495(84)90130-6

[42] Sumithran P , Prendergast LA , Delbridge E *et al* Long‐term persistence of hormonal adaptations to weight loss. N Engl J Med 2011; 365: 1597–1604. 22029981 10.1056/NEJMoa1105816

[43] Townshend T , Lake A . Obesogenic environments: current evidence of the built and food environments. Perspect Public Health 2017; 137: 38–44. 28449616 10.1177/1757913916679860

[44] Xenical^®^ (orlistat 120 mg) . Summary of Product Characteristics, 1998 URL http://www.ema.europa.eu/docs/en_GB/document_library/EPAR_-_Product_Information/human/000154/WC500058428.pdf (accessed 5 April 2017).

[45] US Food and Drug Administration (FDA) . FDA drugs. URL http://www.fda.gov/Drugs/default.htm (accessed 20 December 2017).

[46] EMA . EMA medicines, 2015 URL http://www.ema.europa.eu/ (accessed 20 December 2017).

[47] Alli^®^ (orlistat 60 mg) . Summary of Product Characteristics, 2007 URL http://www.ema.europa.eu/docs/en_GB/document_library/EPAR_-_Product_Information/human/000854/WC500024120.pdf (accessed 20 December 2017).

[48] EMA . Public statement on Acomplia (rimonabant): Withdrawal of marketing authorisation in the European Union (EMEA/39457/2009), 2009 URL http://www.ema.europa.eu/docs/en_GB/document_library/Public_statement/2009/11/WC500012189.pdf (accessed 20 December 2017)].

[49] EMA . Mysimba SmPC. URL http://www.ema.europa.eu/docs/en_GB/document_library/EPAR_-_Product_Information/human/003687/WC500185580.pdf (accessed 12 February 2018)].

[50] EMA . Saxenda SmPC. URL http://www.ema.europa.eu/docs/en_GB/document_library/EPAR_-_Product_Information/human/003780/WC500185786.pdf (accessed 12 February 2018).

[51] National Institute for Health and Care Excellence (NICE) . Obesity, overweight with risk factors ‐ naltrexone‐bupropion (prolonged release) [ID757], In Press 2017 URL https://www.nice.org.uk/guidance/indevelopment/gid-tag486 (accessed 20 December 2017).

[52] EMA . Qsiva: Assessment report. Procedure No. EMEA/H/C/002350/0000. URL http://www.ema.europa.eu/docs/en_GB/document_library/EPAR_-_Public_assessment_report/human/002350/WC500144300.pdf (accessed 12 February 2018).

[53] EMA . Belviq: Assessment report. Procedure No. EMEA/H/C/002597. URL http://www.ema.europa.eu/docs/en_GB/document_library/Application_withdrawal_assessment_report/human/002597/WC500148196.pdf (accessed 12 February 2018)].

[54] James WP , Astrup A , Finer N *et al* Effect of sibutramine on weight maintenance after weight loss: a randomised trial. STORM Study Group Sibutramine Trial of Obesity Reduction and Maintenance. Lancet 2000; 356: 2119–2125. 11191537 10.1016/s0140-6736(00)03491-7

[55] Torgerson JS , Hauptman J , Boldrin MN , Sjostrom L . XENical in the prevention of diabetes in obese subjects (XENDOS) study: a randomized study of orlistat as an adjunct to lifestyle changes for the prevention of type 2 diabetes in obese patients. Diabetes Care 2004; 27: 155–161. 14693982 10.2337/diacare.27.1.155

[56] Eichler HG , Abadie E , Breckenridge A *et al* Bridging the efficacy‐effectiveness gap: a regulator's perspective on addressing variability of drug response. Nat Rev Drug Discov 2011; 10: 495–506. 21720406 10.1038/nrd3501

[57] Rothwell PM . Factors that can affect the external validity of randomised controlled trials. PLoS Clin Trials 2006; 1: e9. 10.1371/journal.pctr.0010009 PMC1488890 16871331

[58] Greenway FL , Fujioka K , Plodkowski RA *et al* Effect of naltrexone plus bupropion on weight loss in overweight and obese adults (COR‐I): a multicentre, randomised, double‐blind, placebo‐controlled, phase 3 trial. Lancet 2010; 376: 595–605. 20673995 10.1016/S0140-6736(10)60888-4

[59] James WP , Caterson ID , Coutinho W *et al* Effect of sibutramine on cardiovascular outcomes in overweight and obese subjects. N Engl J Med 2010; 363: 905–917. 20818901 10.1056/NEJMoa1003114

[60] Li MF , Cheung BM . Rise and fall of anti‐obesity drugs. World J Diabetes 2011; 2: 19–23. 21537456 10.4239/wjd.v2.i2.19 PMC3083904

[61] Apovian CM , Aronne L , Rubino D *et al* A randomized, phase 3 trial of naltrexone SR/bupropion SR on weight and obesity‐related risk factors (COR‐II). Obesity (Silver Spring, Md) 2013; 21: 935–943. 10.1002/oby.20309 PMC3739931 23408728

[62] Hollander P , Gupta AK , Plodkowski R *et al* Effects of naltrexone sustained‐release/bupropion sustained‐release combination therapy on body weight and glycemic parameters in overweight and obese patients with type 2 diabetes. Diabetes Care 2013; 36: 4022–4029. 24144653 10.2337/dc13-0234 PMC3836105

[63] Pi‐Sunyer X , Astrup A , Fujioka K *et al* A randomized, controlled trial of 3.0 mg of Liraglutide in weight management. N Engl J Med 2015; 373: 11–22. 26132939 10.1056/NEJMoa1411892

[64] le Roux CW , Astrup A , Fujioka K *et al* et al, 3 years of liraglutide versus placebo for type 2 diabetes risk reduction and weight management in individuals with prediabetes: a randomised, double‐blind trial. Lancet 2017; 389: 1399–1409. 28237263 10.1016/S0140-6736(17)30069-7

[65] Davies MJ , Bergenstal R , Bode B *et al* Efficacy of Liraglutide for weight loss among patients with type 2 diabetes: the SCALE diabetes randomized clinical trial. JAMA 2015; 314: 687–699. 26284720 10.1001/jama.2015.9676

[66] Wadden TA , Hollander P , Klein S *et al* Weight maintenance and additional weight loss with liraglutide after low‐calorie‐diet‐induced weight loss: the SCALE maintenance randomized study. Int J Obes (Lond) 2013; 37: 1443–1451. 23812094 10.1038/ijo.2013.120

[67] Blackman A , Foster GD , Zammit G *et al* Effect of liraglutide 3.0 mg in individuals with obesity and moderate or severe obstructive sleep apnea: the SCALE sleep apnea randomized clinical trial. Int J Obes (Lond) 2016; 40: 1310–1319. 27005405 10.1038/ijo.2016.52 PMC4973216

[68] Bluher M , Hermansen K , Greenway F *et al* Early weight loss with liraglutide 3.0 mg is good predictor of clinically meaningful weight loss after 56 weeks. Diabetologia 2015; 58: S310 (abstract 645).

[69] National Obesity Observatory . Standard evaluation framework for weight management interventions, 2009 URL http://webarchive.nationalarchives.gov.uk/20160805121933/http://www.noo.org.uk/core/frameworks/SEF (accessed 20 December 2017).

[70] NHS . NHS Digital: Methodology document, 2017 URL https://digital.nhs.uk/catalogue/PUB30142 (accessed 20 December 2017).

[71] Cawley J , Meyerhoefer C , Biener A , Hammer M , Wintfeld N . Savings in Medical Expenditures Associated with reductions in body mass index among US adults with obesity, by diabetes status. Pharmacoeconomics 2015; 33: 707–722. 25381647 10.1007/s40273-014-0230-2 PMC4486410

[72] NHS . NHS Diabetes Prevention Programme and weight management services: eligibility criteria, 2016 URL https://www.england.nhs.uk/wp-content/uploads/2016/07/dpp-wm-service.pdf (accessed 20 December 2017).

[73] Piper C , Marossy A , Griffiths Z , Adegboye A . Evaluation of a type 2 diabetes prevention program using a commercial weight management provider for nondiabetic hyperglycemic patients referred by primary care in the UK. BMJ Open Diab Res Care 2017; 5: e000418. 10.1136/bmjdrc-2017-000418 PMC5706489 29225891

[74] NHS . NHS Diabetes Prevention Programme (NHS DPP), 2016 URL https://www.england.nhs.uk/ourwork/qual-clin-lead/diabetes-prevention (accessed 20 December 2017).

[75] Caterson ID , Finer N , Coutinho W *et al* Maintained intentional weight loss reduces cardiovascular outcomes: results from the Sibutramine cardiovascular OUTcomes (SCOUT) trial. Diabetes Obes Metab 2012; 14: 523–530. 22192338 10.1111/j.1463-1326.2011.01554.x

[76] Lean ME , Leslie WS , Barnes AC *et al* Primary care‐led weight management for remission of type 2 diabetes (DiRECT): an open‐label, cluster‐randomised trial. Lancet 2018; 391: 541–551. 29221645 10.1016/S0140-6736(17)33102-1

[77] O'Neill PM , Birkenfeld AL , McGowan B *et al* A randomised, phase II, placebo‐ and active‐controlled dose‐ranging study of semaglutide for treatment of obesity in subjects without diabetes. Endocr Rev 2018; 39:(Suppl): OR12–OR15.

